# PATIENT EXPOSURE OPTIMISATION THROUGH TASK-BASED ASSESSMENT OF A NEW MODEL-BASED ITERATIVE RECONSTRUCTION TECHNIQUE

**DOI:** 10.1093/rpd/ncw019

**Published:** 2016-06-07

**Authors:** Julien G. Ott, Alexandre Ba, Damien Racine, Nick Ryckx, François O. Bochud, Hatem Alkadhi, Francis R. Verdun

**Affiliations:** 1 Institute of Radiation Physics, CHUV, Lausanne, Switzerland; 2 Institute of Diagnostic and Interventional Radiology, USZ, Zürich, Switzerland

## Abstract

The goal of the present work was to report and investigate the performances of a new iterative reconstruction algorithm, using a model observer. For that, a dedicated low-contrast phantom containing different targets was scanned at four volume computed tomography dose index (CTDI_vol_) levels on a Siemens SOMATOM Force computed tomography (CT). The acquired images were reconstructed using the ADMIRE algorithm and were then assessed by three human observers who performed alternative forced choice experiments. Next, a channelised hotelling observer model was applied on the same set of images. The comparison between the two was performed using the percentage correct as a figure of merit. The results indicated a strong agreement between human and model observer as well as an improvement in the low-contrast detection when switching from an ADMIRE strength of 1–3. Good results were also observed even in situations where the target was hard to detect, suggesting that patient dose could be further reduced and optimised.

## INTRODUCTION

Over the past decade, the radiation dose delivered to patients via diagnostic X-ray imaging has continuously increased until today, where it reaches 25 % of the accumulated man-made and natural radiation contributions. Among that 25 %, computed tomography (CT) raises a particular concern, since this imaging modality represents in Switzerland for example 68 % of the collective dose, yet only 8 % of the number of examinations^(1)^. In this context, CT manufacturers have developed new strategies like iterative reconstruction (IR) algorithms in order to ensure that the benefits–risk ratio remains in favour of the patient. This new technology certainly improved the clinical practice^(2)^, but it has also led to drastic changes in image perception. Thus, ensuring an adequate level of image quality while keeping patient's exposure as low as reasonably achievable constitutes a new challenge to be addressed. The use of task-based image quality assessment method could represent an efficient way to perform this optimisation scheme^(3, 4)^. Therefore, the goal of this study was to report and investigate the performances of a new IR technique using a model observer that mimics human detection of low-contrast targets: the channelised Hotelling observer (CHO) model.

## MATERIALS AND METHODS

### Data acquisition

A dedicated low-contrast phantom (QRM, Moehrendorf, Germany), mimicking the attenuation produced by a patient's chest, was used. The phantom could embed two different custom-made modules in its middle: a homogeneous modulus and another containing low-contrast spherical targets of 6 and 8 mm in diameter with contrast levels of 10 and 20 HU at 120 kVp.

Data acquisition was performed at the University Hospital Zurich on a third-generation dual-source 192-slice CT scanner (SOMATOM Force, Siemens Healthcare, Erlangen, Germany). A tube voltage of 120 kVp, a 300 mm display field of view (DFOV), a 512 × 512 matrix size and 2.0-mm-thick slices, which were reconstructed every 1.0 mm, were used. Acquisitions were performed in the helical mode with a pitch of 0.98. Four dose levels [1.0, 3.5, 8.0 and 15.0 mGy expressed in a volume computed tomography dose index (CTDI_vol_) phantom of 32 cm in diameter] were investigated, using the procedure described in the IEC 60601-2-44^(5)^ to measure the CTDI_vol_. The phantom was scanned 20 times for each condition. Reconstructions were performed using the Siemens advanced model iterative reconstruction (ADMIRE) with strength levels 1 and 3. On the machine, users can choose ADMIRE levels ranging from 1 to 5, with level 1 being closest to the image impression of traditional, filtered back-projection, and level 5 showing the strongest noise reduction. In the end, 32 different categories were obtained (four dose levels, two ADMIRE levels, two contrast levels and two target sizes). From these sets of acquisitions, regions of interest (ROIs) of 41 × 41 pixels (0.59 mm pixel size) containing the centred targets were extracted. For each category, 100 images containing a signal (20 scans × 5 targets with identical size and contrast in the phantom) and 1000 images with only noise were extracted. The ROIs that contained noise only were extracted from the homogeneous modulus, whereas the ROIs containing the signals came from the low-contrast modulus. This methodology enabled holding the same position on the (*x*,*y*) plane for both signal and noise ROIs, in turn enabling solving the noise stationarity problem.

### Human observer

In the human observer experiment, three medical physicists took part in four alternative forced choice (4-AFC) experiments in order to yield a percentage of correct responses (PC) indicating how well they managed to detect the signals. The 4-AFC experiment consisted in selecting the signal-present image in a batch of three signal-absent images and one signal-present image, which were presented together in a randomised order. All observers were blinded to the CT acquisition and reconstruction conditions and began their test with a training session that was made of images acquired at high dose level. They were then asked to make decisions for all 32 categories acquired. The previously made acquisitions provided 100 signal-present ROIs and 1000 signal-absent ROIs for each category. ROIs among those data were selected randomly and used for the 4-AFC tests. For each observer and category, every answer to the 100 trials was stored and compared with the correct response, allowing the computation of the PC.

### CHO model observer

A model observer enables to predict the detection of low-contrast signals by calculating a scalar response called the decision variable and denoted by *λ_i_*. This parameter is given by

(1) $$\lambda_{i}=w^{T}\times g_{i},$$

where *w* is the template of the model observer, and *g**_i_***is the analysed ROI (*i* = *n* or *i* = *s* represents signal-absent or signal-present hypothesis, respectively).

The CHO model used in this study is an anthropomorphic model observer also including preprocessing of the image by a set of channels that enhance some spatial frequencies^(6, 7)^. The template *w*_CHO_ of this model is obtained as explained in the following part (extensive details can be found elsewhere^(8–11)^):

(2) $$w_{\mathrm{CHO}}={\left[\frac{1}{2}(K_{\mathrm{cs}}+K_{\mathrm{cn}})\right]}^{-1}(\overset{-}{g_{\mathrm{cs}}}-\overset{-}{g_{\mathrm{cn}}}),$$

where

(3) $$K_{\mathrm{cn}}=U^{T}K_{n}U\;\text{and}\;K_{\mathrm{cs}}=U^{T}K_{s}U.$$

In this equation, *K_n_* and *K_s_* are the covariance matrix calculated, respectively, from the signal-absent and signal-present data, and ***U*** is the matrix representation of the channel filters described more extensively below.

In Equation 2, $\overset{-}{g_{\mathrm{cs}}}$ and $\overset{-}{g_{\mathrm{cn}}}$ are the means of the channel outputs under a signal-present and signal-absent hypothesis and can be estimated according to

(4) $$\overset{-}{g_{\mathrm{cs}}}=U^{T}\overset{-}{g_{s}}\;\text{and}\;\overset{-}{g_{\mathrm{cn}}}=U^{T}\overset{-}{g_{n}}.$$

The employed set of channels is called dense difference of Gaussian (DDoG) channels and was described by Abbey and Barrett in 2001^(12)^. It includes 10 channels for which the radial frequency profile of the *j*th channel is given by

(5) $$U_{j}(\rho )=\text{exp}\left[-\frac{1}{2}{\left(\frac{\rho }{W\sigma_{j}}\right)}^{2}\right]-\text{exp}\left[-\frac{1}{2}{\left(\frac{\rho }{\sigma_{j}}\right)}^{2}\right],$$

where *ρ* is the spatial frequency, *W* = 1.67 defines the bandwidth of the channel, and *σ_j_* is the standard deviation of the *j*th channel. Each *σ_j_* value is defined by the equation $\sigma_{j}=\sigma_{o}\alpha^{j}$ with *σ_o_* = 0.005 and *α* = 1.4^(12)^.

The decision variable can then be calculated by injecting the channel output of the ROI (denoted by *g*_ci_) and the template ***w***_CHO_ in Equation 1:

(6) $$\lambda_{i}=w_{\mathrm{CHO}}^{T}\times g_{\mathrm{ci}}.$$

The PC was obtained using the CHO model to perform 4-AFC tests on the acquired images. The value of the decision variable was used to determine which of the four images contained the signal (the highest value of *λ* is supposed to be the signal-present image). Then, the results of the model observers were compared with the truth in order to enabling the computation of PCs.

### Uncertainty estimation

The uncertainties of the models' results were estimated by performing bootstrap^(13)^. According to the bootstrap method and the set of 100 signal-present ROIs, the 4-AFC test was performed 150 times for each category, leading to 150 values of PC. Then, the mean and standard deviation of the 150 values obtained were computed in order to determine the final mean PC value as well as its standard deviation for each acquisition condition. This allowed to estimate a 95 % confidence interval. The uncertainties for the human observers were calculated using the results of the three different observers. For each category, the mean PC value of the observers and its standard error were calculated in order to display a 95 % confidence interval.

## RESULTS

**Figure 1.**
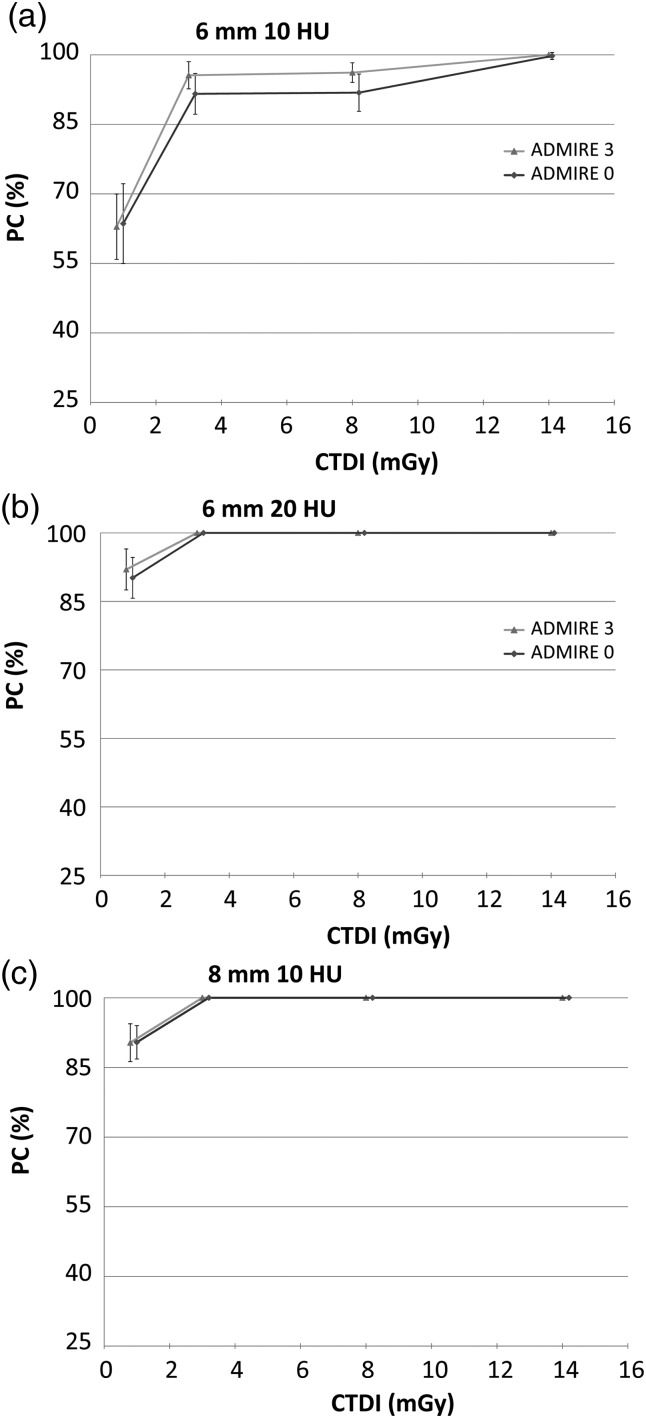
Results of the CHO model observer (in PC, obtained by performing 4-AFC tests).

**Figure 2.**
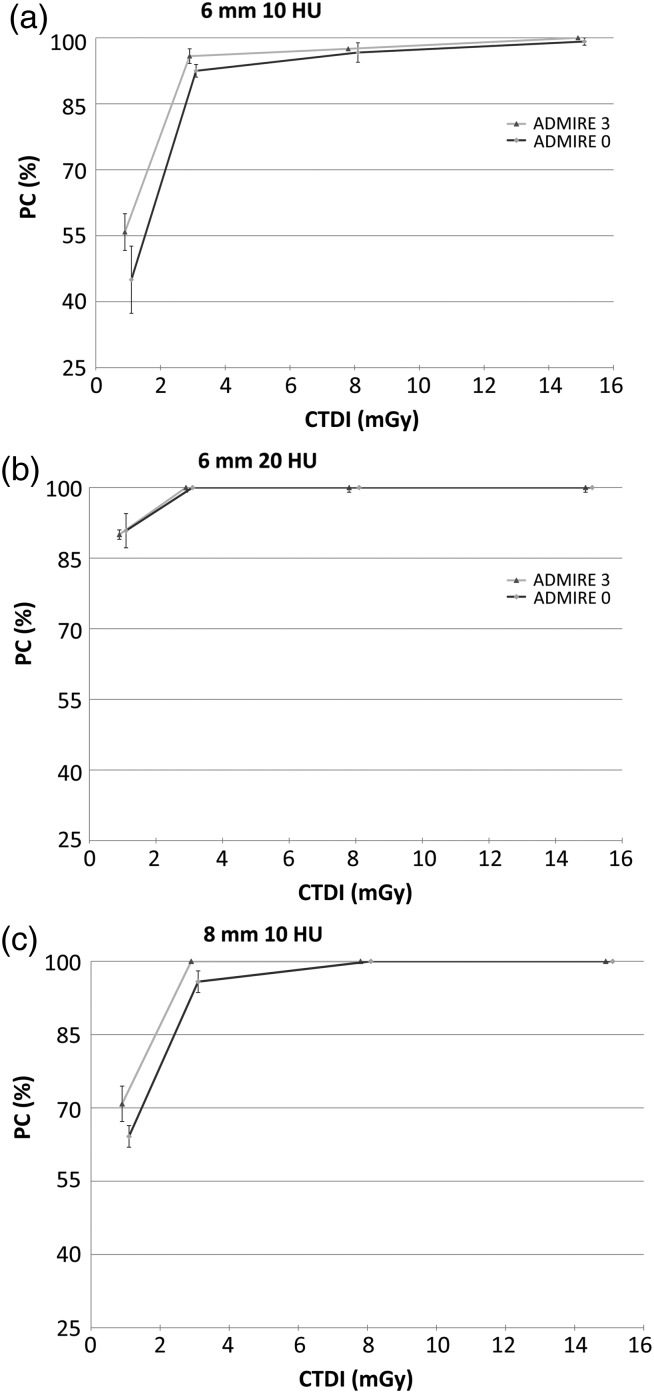
Results of the three human observers (in PC, obtained by performing 4-AFC tests).

In this section, the qualities of the images obtained using the ADMIRE algorithm with strength levels 1 and 3 are assessed through the performances of model (Figure 1) and human observers (Figure 2).

Results of the CHO model with the DDoG channels suggest that both strength levels exhibited PCs in the same range with an increase of a few per cent in the results when switching from strength level 1 to 3. This trend was, however, only observed for certain signals and dose range, namely the lowest contrast (10 HU) associated to low dose levels (1.0 and 3.5 mGy).

Human observers exhibited results very similar to the ones obtained with the CHO model in terms of PC values. The results also suggest that the use of higher ADMIRE strength level is useful to improve the detection of small-size and low-contrast signal under low CTDI_vol_. On top of that, it was observed that the largest signal size (8 mm) with the highest contrast (20 HU) corresponded to a trivially easy task with PC always equal to 100 % no matter which dose and ADMIRE level was employed. Those results remained true for both human and model observers and are therefore not presented in Figures 1 and 2.

Model and human observers exhibited a great adequacy in their results, both of them indicating that the use of a higher ADMIRE level enhances the detection when working under conditions where the signal is hard to detect. It was also witnessed that the PC increases with the given dose until it reaches an asymptote and that this asymptote is reached faster when using higher ADMIRE strength level. Furthermore, the results enlighten that no more dose increase is needed once an amount of ∼4 mGy is reached because the performance of the human observer is already almost maximal at this point.

## DISCUSSION

The goal of the present investigation was to determine if the use of the ADMIRE algorithm at different strength could lead to a high detection performance, therefore allowing a further dose reduction in the clinical practice. The results showed that the CHO model with DDoG channels gave coherent results since it reproduced the behaviour of humans very well and under a wide range of conditions and signal contrasts. The model seems to be very efficient in low-contrast detection and even sometimes overestimates human results for low contrast and low doses. These results are coherent with recent studies from Leng and Yu^(14)^ which showed the efficiency of the CHO model for the low-contrast detection. Also, both model and human observers reported a visible improvement in the low-contrast detection when increasing the ADMIRE strength. This trend was observed when working at low dose levels (<4 mGy) for all signal types. Indeed, when working at higher dose levels, the PCs always reached 100 %, letting no room for improvement. In the end, the use of ADMIRE makes it possible to diminish the dose without losing information in the image. Indeed, the PC results obtained in the study reach very high or perfect values for almost every acquisition condition and signal, indicating that a dose reduction without impacting the detection performance would be possible.

However, some limitations of the present study have to be underlined. First among them, the number of images acquired may be considered as low since not enough ROIs were disposed to separate them in two exploitable data sets. Usually, a first set is used for the computation of the covariance matrix in the determination of the template of the CHO model, while the second data set is used for the computation of the PCs. However, it is worth to underline that Barrett and Myers^(15)^ who studied this problem concluded that using one single set of data remained a reliable way to proceed. Moreover, performing a limited number of acquisitions (20 scans for each acquisition condition in the present case) allowed to reduce the operating time of the device, which is appreciable when working in a clinical environment. The second limitation faced is that the paradigm worked with (when the signal, location and background are exactly known) was simplified and therefore different from real anatomical conditions. The results could nevertheless be used in order to assess the performances of the tested IR algorithms, but it is worth to mention that there is room for a more complex study on the subject.

## CONCLUSION

Nowadays, assessing CT image quality cannot be done with image space metrics anymore. Moreover, evidence indicates that frequency metrics should not be used either when working with IR. However, the task-based tool used in this investigation (CHO model observer associated to the DDoG channels) successfully demonstrated its ability to reproduce the human's response in a low-contrast detection task, thus establishing its reliability for image quality assessment.

The results obtained with this tool revealed that the ADMIRE algorithm led to high PCs even in situations where the target was harder to detect (i.e. low CTDI_vol_ and contrast level). Also, using higher ADMIRE strength led to PC improvement, particularly in the low CTDI_vol_ range. Therefore, using those benefits to keep the image quality equivalent to what was previously obtained would enable to spare some delivered dose.

All those elements suggest that the patient dose could be further optimised and reduced thanks to the use of the ADMIRE algorithm and this new CT unit.

## FUNDING

This work was partly supported by a grant from the Swiss National Science Foundation (No. 320030-140995). Funding to pay the Open Access publication charges for this article was provided by the institute of radiation physics from Lausanne.
